# Patients’ Self-Reported Recovery After an Environmental Intervention Aimed to Support Patient’s Circadian Rhythm in Intensive Care

**DOI:** 10.1177/19375867211001541

**Published:** 2021-03-23

**Authors:** Marie Engwall, Göran Jutengren, Ingegerd Bergbom, Berit Lindahl, Isabell Fridh

**Affiliations:** 1 387496 Department of Health Sciences, University West, Trollhättan, Sweden; 2 Faculty of Health and Welfare, Østfold University College, Halden, Norway; 3 Institute of Health and Caring Sciences, Sahlgrenska Academy, 3570 Gothenburg University, Sweden; 4 Department of Health Sciences, Faculty of Medicine, 5193 University of Lund, Sweden; 5 Faculty of Caring Sciences, Work Life and Social Welfare, 1802 University of Borås, Sweden; 6 Department of Anesthesiology, Surgery, and Intensive Care, Sahlgrenska University Hospital, Gothenburg, Sweden

**Keywords:** ANOVA, circadian rhythm, environment, intensive care unit (ICU), lighting, longitudinal, recovery, sleep, questionnaire

## Abstract

**Background:**

Patients in intensive care units (ICUs) are among the most vulnerable, and they require support to start their recovery. The design of the patient area in the ICU can play a prominent role in both the quality of care and patients’ recovery. The lighting environment has the opportunity to restore and strengthen the natural human circadian rhythm and health.

**Aim:**

To evaluate patients’ self-reported recovery after being cared for in an ICU room rebuilt according to evidence-based design principles that promote recovery.

**Method:**

An intervention was set up in a two-bed patient room including a cycled lighting system. Self-reported recovery was reported at 6 and 12 months after discharge. Data were analyzed using a 2(mechanically ventilated, nonmechanically ventilated) × 2(intervention room, ordinary room) analysis of covariance (ANCOVA) and 2(male, women) × 2(intervention room, ordinary room) ANCOVA.

**Results:**

Data from the different rooms showed no significant main effects for recovery after 6 months, *p* = .21; however, after 12 months, it become significant, *p*. < .05. This indicated that patient recovery was positively influenced for patients cared for in the intervention room (*M* = 8.88, *SD* = 4.07) compared to the ordinary room (*M* = 10.90, *SD* = 4.26). There were no interaction effects for gender or if the patients had been mechanically ventilated either at 6 or 12 months’ postdischarge.

**Conclusions:**

A cycled lighting system may improve patient self-reported recovery after ICU care; however, more research on the topic is needed.

The physical environment of intensive care units (ICUs) should be considered as a part of patient care. They should be designed to support and promote healing and recovery of critical illnesses or injuries. The design of the patient area in the ICU plays a prominent role for both the quality of care and patients’ recovery (Olausson et al., 2013; Rashid, 2014; Zborowsky & Hellmich, 2011). This article presents the results collected through a questionnaire investigating patients’ self-reported recovery after being cared for in an ICU. The main purpose of this study was to investigate whether a lighting design of the ICU patient room had an impact on patients’ longer term recovery at 6 and 12 months’ postdischarge.

## The ICU Environment

Patients cared for in an ICU are among the most vulnerable and need all the support they can be offered to start their healing process. Researchers stated that to have a successful ICU design, there should be cooperation between ICU staff, architects, and designers (Chambers & Bowman, 2011). Sometimes, there are opposing demands from patients, staff, and relatives to consider when designing the ICU environment taking both functionality and healing aspects into account. For example, at night, staff prefer bright lighting to be able to perform medical tasks faster and more efficiently while patients prefer it darker. This situation makes the process challenging and in need of a professional approach (Altimier, 2004; Bazuin & Cardon, 2011). Starting in the 1990s, research findings concerning physical environments and their effects on the users’ health were brought into the concept of evidence-based design (EBD; Peavey & Vander, 2017; Ulrich et al., 2010). Healing environments are designed based on theories of EBD to focus on the impact of the space on peoples’ healing and recovery (Bazuin & Cardon, 2011; Schreuder et al., 2016).

***Patients cared for in an ICU are among the most vulnerable and need all the support they can be offered to start their healing process. Researchers stated that to have a successful ICU design, there should be cooperation between ICU staff, architects, and designers.***

The lighting environment and its effect on adult ICU patients’ health and recovery have not yet been fully investigated. There are studies that have investigated lighting levels (Bani Younis et al., 2019; Engwall et al., 2014) and environmental noise and light reduction programs (Patel et al., 2014; Yaşar et al., 2017). However, research evaluating lighting interventions which aimed to support patients’ sleep, circadian rhythm (CR), and recovery are rare.

## The Connection Between ICU Environment and Recovery From Severe Illness or Injury

The environment in the ICU is one factor for sleep deprivation which is known to be a stressor for patients (Elliott et al., 2013). Noise is a persistently reported sleep-disruptive factor (Farshidpanah et al., 2017). Furthermore, the lighting levels in the ICU are frequently low in daytime and high at nighttime, compared to recommendations reported in a review (Engwall et al., 2014). Light, at right time, at the appropriate level, and with spectral distributions, has the opportunity to restore and strengthen the natural human CR by communicating with the suprachiasmatic nucleus in the anterior hypothalamus (Bonmati-Carrion et al., 2014; Figueiro et al., 2019; Wright et al., 2013). This nucleus works as an internal clock for bodily rhythms, and it controls the systems by giving signals to hormone-producing glands to release more or fewer hormones such as melatonin (Hastings et al., 2007). The lighting levels in the ICU room may not be able to synchronize with a patient’s CR (Gronfier et al., 2007). Disruptions in the CR occur for most patients cared for in the ICU (Gehlbach et al., 2012; Knauert et al., 2015) especially for those suffering from sepsis (Mundigler et al., 2002). To give the patients the opportunity to recover, it is important to provide interventions in the environment that supports their normal CRs (Brainard et al., 2015). The CR impacts most bodily processes such as sleep, wakefulness, metabolism, hormone release, cardiovascular activation, renal filtration (Hastings et al., 2007), temperature (Khalsa et al., 2000), and the immune system (Scheiermann et al., 2013).

***Light, at right time, at the appropriate level, and with spectral distributions, has the opportunity to restore and strengthen the natural human CR by communicating with the suprachiasmatic nucleus in the anterior hypothalamus.***

***To give the patients the opportunity to recover, it is important to provide interventions in the environment that supports their normal CRs.***

## Consequences for Patients’ Health Connected to ICU Care

A negative correlation between light and sound levels with quality of sleep in the ICU was reported in a study by Bani Younis and colleagues (2019). According to Farshidpanah et al. (2017), sleep problems can continue for a long time after discharge from the ICU and may inhibit recovery. Delirium, with symptoms like hallucinations, can result from sleep deprivation or can be the cause of it. The prevalence of delirium ranges from 64.4% (Shehabi et al., 2010) to 80% (Page & Ely, 2011) in mechanically ventilated ICU patients. Patients who develop delirium have also been shown to have higher mortality rates (Heymann et al., 2010), longer hospital stays (Shehabi et al., 2010), and long-term cognitive impairments (Girard, 2010) compared to patients who do not develop delirium. Moreover, former ICU patients are at a higher risk for developing cognitive, physical, and psychological sequelae, called postintensive care syndrome (Khan et al., 2015; Bryant & McNabb, 2019). It has been reported that after ICU care, nearly 50% of the patients displayed neuromyopathy (Stevens et al., 2007), and in another study, it was found that 23% and 28% of the patients studied, 6 months after being discharged, had symptoms of depression and anxiety, respectively (Castillo et al., 2016). Furthermore, Rovatti et al. (2012) reported that 24.4% of the patients suffered from symptoms from posttraumatic stress, and 70% displayed cognitive deterioration in their study (Desai et al., 2011).

## Patients’ Recovery and Follow-Up After the ICU Period

Only patients are able to describe themselves as fully recovered or not. Like the concept of health, recovery is an overall concept including physical, psychological/mental, and existential dimensions of life (Bergbom, 2008; King et al., 2019). The recovery process for patients who have received care in the ICU, often due to organ failure, is dependent on time, and a period of at least 2 years has been mentioned in the literature for patients’ full recovery after receiving care in the ICU. However, the length of recovery can also be affected by other factors such as comorbidities, age, and other life circumstances (Bergbom, 2008).

Patients’ recovery and health conditions after the ICU are often measured by validated instruments such as the Medical Outcomes Study 36-item Short Form (Ware & Sherbourne, 1992), European Quality of Life–Five Dimensions (EuroQol-5D; Brooks, 1996), Sickness Impact Profile (Bergner et al., 1981), and the Nottingham Health Profile (Hunt et al., 1985). They are commonly used, one or a few together, while measuring patients’ quality of life (QoL).

As seen, patients’ recovery after being cared for in the ICU is investigated using several instruments with a wide variety of questions. An instrument with fewer questions to evaluate patients’ self-reported recovery, especially after critical care, was desirable to avoid overwhelming participants by asking them to participate in research with extensive questioning. Our questionnaire was created based on the previous research concerning patients’ recovery process after being cared for in the ICU but in a more simplified manner (Bergbom, 2008).

Previous research has shown that the recovery period after acute disease differs between men and women. Women cared for in the ICU reported higher psychological problems, 14 months after discharge from the ICU measured with Impact of Event Scale for posttraumatic stress and Hospital Anxiety and Depression Scale for anxiety and depression (Schandl et al., 2012). Furthermore, women who have undergone acute myocardial infarctions reported worse recovery after 1 month measured by different scales including the EuroQol-5D (Xu et al., 2015). In an interview study, it was reported that patients’ recovery after psychiatric care included both gender differences and gender stereotypes (Schön, 2013). Also, differences in recovery between patients with or without mechanical ventilation and gender were found, and these aspects are important to include when investigating patients’ recovery.

There has been substantial development in technical, medical, and pharmacological treatments for patients in the ICU. However, the environment in the patients’ rooms has been neglected and not developed to the same extent to be able to support health and recovery. Technical equipment with disturbing noise and light around the clock is dominating the rooms today. It is, therefore, essential to provide an environment that supports patients’ health and their ability to recover. It is important to investigate which, and to what extent, environmental factors can influence recovery.

## Aim

The aim of this study was to evaluate patients’ self-reported recovery after being cared for in an ICU room rebuilt according to EBD principles or an ordinary room. More specifically, the following research questions were investigated: (1) Are there any differences in self-reported recovery and between patients cared for in the rebuilt and cycled lit room compared to patients cared for in an ordinary room? (2) Does mechanical ventilation and gender affect the self-reported recovery when combined with a rebuilt and cycled lit room compared to patients cared for in an ordinary room?

## Method

**Figure 1.**
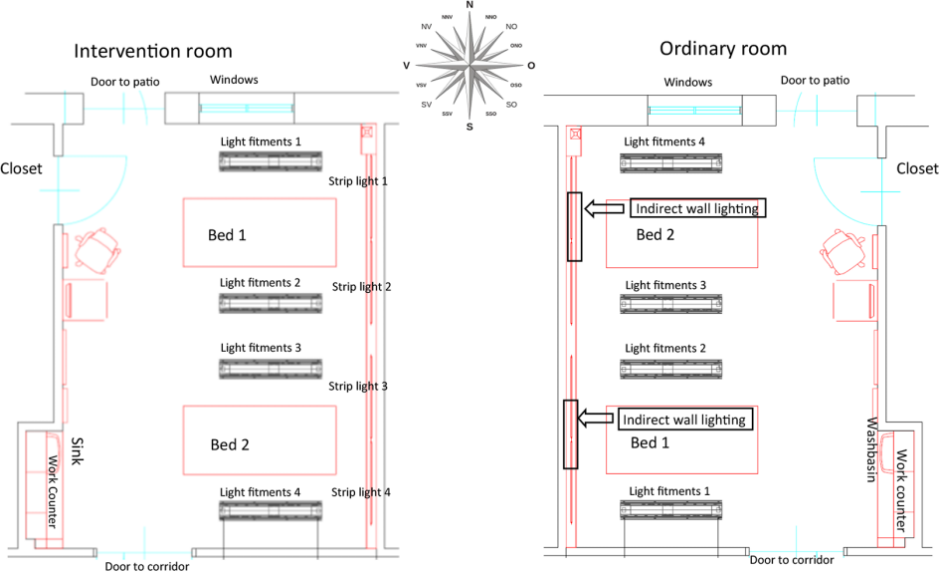
Plan of the intervention room and the ordinary room (Engwall et al., 2014).

The present study was part of a larger research program concerning the healthcare environment in the ICU and its effect on health, well-being, and recovery (Engwall et al., 2014, 2015, 2017). The intervention was set up in a two-bed patient room in accordance to evidence-based principles including a changed lighting system and different ceiling and interior design (Engwall et al., 2015). An ordinary designed two bedroom was used as the control (ordinary room; Figure 1).

Clinical variables were collected from the patient medical record and reported in Table 1. The assessment of predicted mortality and severity of illness was measured by the Simplified Acute Physiology Score 3 (SAPS; Moreno et al., 2005). The Confusion Assessment Method for the ICU (Gélinas et al., 2018) was used to monitor patients for the development of delirium, and patients were reported as positive or negative for delirium.

**Table 1.** Patient Characteristics in the Two Conditions.

	Intervention Room			Ordinary Room			*p* Value
Characteristics	Not Mechanically Ventilated^a^	Mechanically Ventilated	Total	Not Mechanically Ventilated^b^	Mechanically Ventilated	Total	
*N* (%)^c^	21 (46.7%)	24 (53.3%)	45	25 (44.6%)	31 (55.3%)	56	.50
Mean age (*SD*)^d^	58 (19)	67 (8)	63 (15)	63 (15)	64 (11)	64 (13)	.72
Number of men (%)^c^	13 (61.9%)	15 (62.5%)	28 (62.2%)	9 (36%)	18 (58.1%)	27 (48.2%)	.32
Number of women (%)^c^	8 (38.1%)	9 (37.5%)	17 (37.8%)	16 (64%)	13 (41.9%)	29 (51.8%)	.59
Mean SAPS 3 (*SD*)^d^	42 (11)	57 (9)	50 (15)	47 (13)	57 (14)	49 (19)	.81
Number of CAM-ICU-positive (%)^e^	0 (0%)	7 (19.2%)	7 (19.2%)	2 (9.5%)	5 (20.0%)	7 (15.2%)	
Reason for ICU Admission			*n* (%)			*n* (%)	
Pulmonary/respiratory			13 (28.9)			9 (16.1)	
Cardiac—medical			2 (4.4)			3 (5.4)	
Cardiac—surgical			8 (17.8)			8 (14.3)	
Sepsis/infection			6 (13.3)			11 (19.5)	
Internal medicine			12 (26.8)			13 (23.2)	
Trauma			2 (4.4)			2 (3.6)	
Other surgical			2 (2.2)			3 (5.4)	
Other			1 (2.2)			7 (12.5)	

*Note.* ICU = intensive care unit; CAM-ICU = Confusion Assessment Method for the ICU; SAPS = Simplified Acute Physiology Score.

^a^ Mean SAPS 3, *n* = 20, due to missing data.^b^ Mean SAPS, *n* = 21, due to missing data.^c^ *p* Values are based on χ^2^ comparisons of frequencies.^d^ *p* Values are based on *t*-test comparisons of mean values.^e^ Due to missing data, the total number of CAM-ICU-positive was *N* = 83, whereas *n* = 37 in the intervention room (*n* = 19 not mechanically ventilated, *n* = 18 mechanically ventilated) and *n* = 46 in the ordinary room (*n* = 21 not mechanically ventilated, *n* = 25 mechanically ventilated).

## Setting

**Figure 2.**
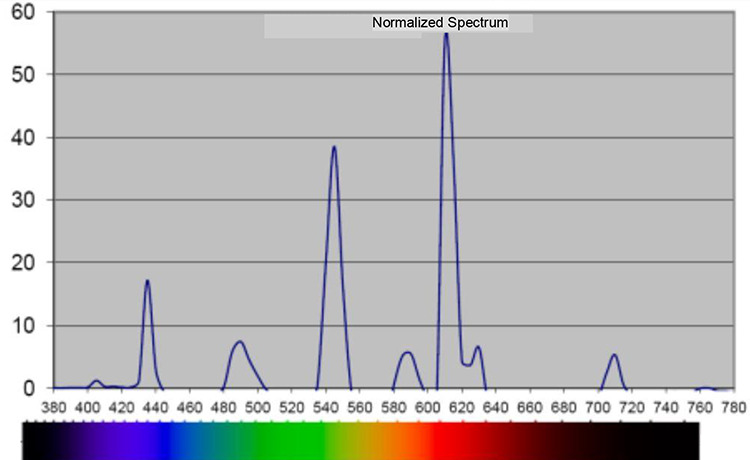
Spectral distribution, in nanometer, during night, 21:30–07:00 in the intervention room. *Note*. Measured 1.1 m above the floor with 120 Spectometer AvaSpec 2048-USB2, software AvaSoft Version 7.5.3.

**Figure 3.**
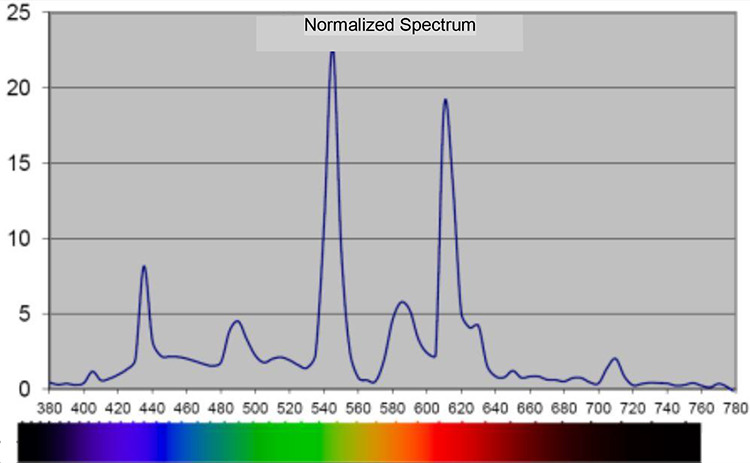
Spectral distribution, in nanometer, during day, 10:30–13:00 and 17:00–18:00, in the intervention room. *Note*. Measured 1.1 m above the floor with 120 Spectometer AvaSpec 2048-USB2, software AvaSoft Version 7.5.3.

The study was conducted at a standard ICU ward with eight beds in a Swedish hospital. The intervention that was set up in 2010 aimed to create a more healing environment to support health and recovery for critically ill patients (Engwall et al., 2014, 2015). A major change in the intervention room was the lighting system, a programmable Digital Addressable Lighting Interface lighting system with Router DIGIDIM 910, software designer with a 9242 touch screen (Helvar, Espoo, Finland). The staff were able to control the lighting system manually for patient care and emergencies through a panel on the front of the lighting armature in the ceiling (hanging approximately 50 cm below the ceiling; Fagerhult, Habo, Sweden) or by the 5.7″ touch screen on the wall (Capax display, Copenhagen, Denmark). The lighting system was constructed to support the patients’ sleep, rest, and CRs by mimicking the rhythm of natural light. It can be described in four parts. (1) The system was automatically controlled by software. This guaranteed an autonomous rhythm, brightness, color temperature, and spectral distribution of the lighting. (2) The brightness was changed throughout the day and night with 14 different light sequences. (3) The location of the light fittings supported the natural rhythm of light. In the morning and in the evening, the light (fluorescent lamps, 2700 K) came from a low position from the wall, behind the headboard. At noon, it came from the ceiling, from armatures (fluorescent lamps, 2700 K and 6500 K), imitating the position of the sun. At night, lights only came from the LED lighting on the floor to ensure the staff and patients could orientate themselves in the room. (4) The color temperature of the light, measured in Kelvin, varied during the day. This was due to a combination of the light sources’ color temperature, 2700 K and 6500 K, respectively, and how much power was given out from the different lighting sources. Measurements of illuminance levels, color temperature, and spectral distribution are reported in Table 2 and Figures 2 and 3.

**Table 2.** Schedule Over Lighting Locations, Levels, and Colors in the Intervention Room.

Light Setting No.	Time	Locations of Lighting	Levels % 2700 K	Levels % 6500 K	Locations of Lighting	Levels % 2700 K	Locations of Lighting	Levels % 2700 K	Illumination Levels in Lux in Horizontal Plane at the Patients’ Head
0	07:00–08:00	Ceiling	10	5	Wall	15			58
1	08:00–10:00	Ceiling	100	100	Wall	100	Floor	100	615
2	10:00–10:30	Ceiling	70	100					450
3	10:30–13:00	Ceiling	37	85					330
4	13:00–15:00	Ceiling	23	52					210
5	15:00–17:00	Ceiling	70	100					450
6	17:00–18:00	Ceiling	37	85					330
7	18:00–19:00	Ceiling	23	52					210
8	19:00–20:00	Ceiling	14	15	Wall	1			81
9	20:00–20:45	Ceiling	10	5	Wall	15			58
10	20:45–21:00	Ceiling	6	1	Wall	7			30
11	21:00–21:15	Ceiling	3		Wall	1			12
12	21:15–21:30	Ceiling	1		Wall	1			8
13	21:30–07:00						Floor	80	2

*Source*. Engwall et al. (2014). *Note*. Measured in horizontal plane at the patients’ head in January 2012.

Other components that were changed in the intervention room were the ceiling and the textiles. This resulted in a slight better acoustic environment with regard to distinctness of speech and less reverberation (Johansson, 2014). Moreover, the interior design was created by the idea that a calm surface would mentally relax the patients (Lindahl & Bergbom, 2015).

**Figure 4.**
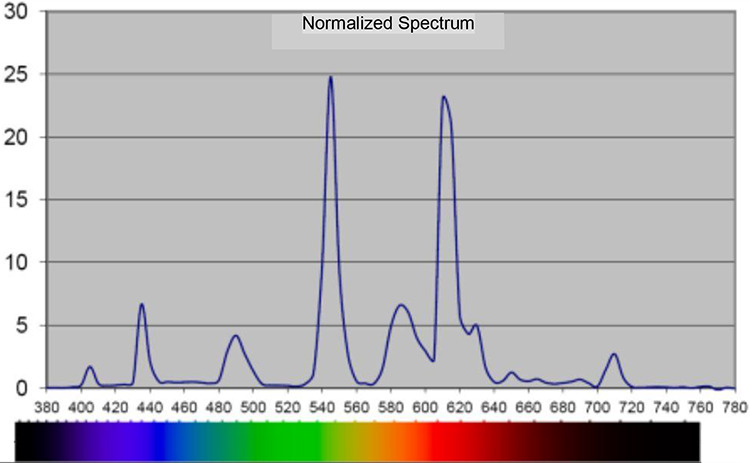
Spectral distribution, in nanometer, indirect wall lighting in the ordinary room. *Note*. Measured 1.1 m above the floor with 120 Spectometer AvaSpec 2048-USB2, software AvaSoft Version 7.5.3.

**Figure 5.**
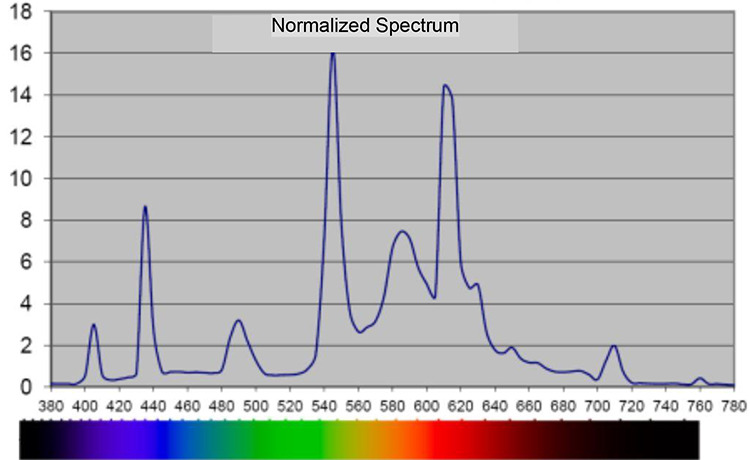
Spectral distribution, in nanometer, indirect wall lighting and fluorescent lighting in the ceiling in the ordinary room. *Note*. Measured 1.1 m above the floor with 120 Spectometer AvaSpec 2048-USB2, software AvaSoft Version 7.5.3.

The lighting system in the control room (Table 3) was set up in 1992 and consisted of light fittings in the ceiling (fluorescent lamps), which shined downward. Furthermore, indirect wall lighting (fluorescent lamps) was located behind the patients’ beds, and a night lamp (low-energy lamps) was hanging from the ceiling at the end of each patient bed. Normally, only the wall lighting was switched on during the daytime; however, sometimes the wall lighting was used in combination with the light fittings in the ceiling. At night, the night lamp or the wall lighting was used. The lighting was controlled manually by the staff due to their or the patients’ demands or wishes. Measurements of illuminance levels and spectral distribution are reported in Table 3 and Figures 4 and 5.

**Table 3.** Illumination Settings and Levels in the Ordinary Room.

Lighting Sources in Control Room	Illumination Levels in Lux
Lightings at wall	147
Lightings in ceiling and walls	810
Night lamps	0.7

*Source*. Engwall et al. (2015). *Note*. Measured in horizontal plane at the patients’ head in January 2012.

## Participants and Procedure

Discharged patients, admitted in the ICU between August 2012 and May 2014 and cared for in the intervention or the ordinary room, were asked to participate (see Figure 3). During their ICU stays, patients had been assigned to one of the two rooms according to the ward’s ordinary patient flow. If there were vacant beds in both rooms, patients were assigned randomly. In the case of staff limitations, the patients’ health conditions and needs to accommodate medical equipment determined the room allocation. The inclusion criteria were (1) competence in the Swedish language and (2) over the age of 18. The exclusion criteria were (1) severe brain injuries, (2) dementia, and (3) blindness. All patients who met the inclusion criteria were included in the study, except for those who met the exclusion criteria or were missed for various reasons (see Figure 3). The size of the sample was not statistically calculated.

**Figure 6.**
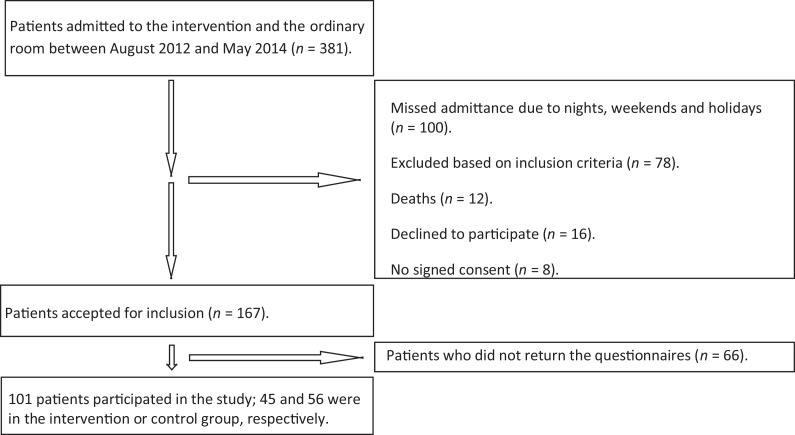
Flowchart describing the patient inclusion process.

## The Questionnaire

**Figure 7.**
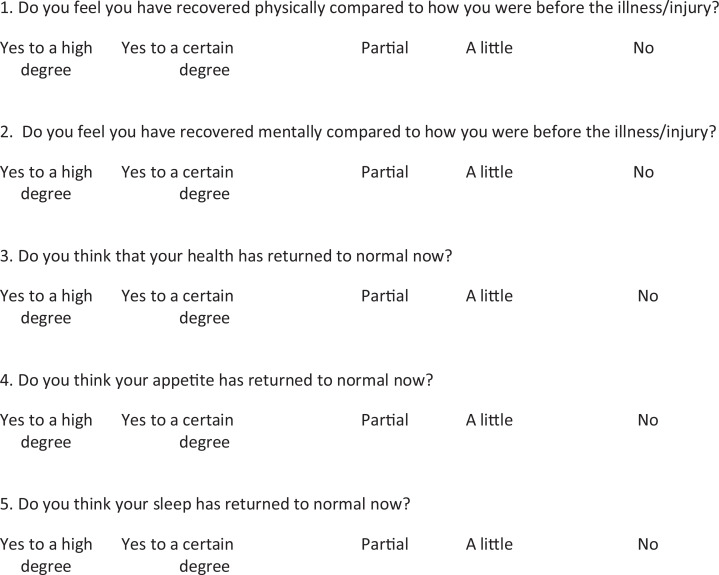
Questionnaire concerning patients’ self-estimated recovery after intensive care.

Five questions from the Patient Self-report Recovery Questionnaire (Bergbom et al., 2018) were used to measure the degree of recovery in terms of physical and mental health (see Figure 7). Patients responded to a 5-point Likert-type scale, ranging from *yes, to a high degree* to *No*. The questionnaire was validated in accordance to scientific principles. A factor analysis was run, and all five Likert-type questions were found to map onto one single factor, which explained 49.92% of the total variance of the model. The reliability was tested, and Cronbach’s α was .80 for 6 months, and .82 for 12 months, indicating high reliability. The validity for the new dependent variable, recovery, was correlated with a question concerning patients’ general health state (“Do you have symptoms of something that are related to the disease/injury?”), with the same response scale as for the recovery measure. The result indicates acceptable criterion validity, *r* = .51, *p* < .01, *N* = 164.

## Data Collection

Two questionnaires were sent out to patients by mail; one at 6 and the other at 12 months after ICU discharge. Patients were asked to give their responses about their recovery after having received ICU care in one of the two rooms due to a severe illness or injury. All questionnaires were marked with a number, and no personal identification numbers or names were used to ensure confidentiality. An information letter about the study was added to the questionnaire with information about how to contact the researcher and returning the questionnaire by mail within a week. A prestamped envelope was also included, so participants did not incur any costs. A reminder, along with another questionnaire, was sent out to those who had not responded after 2 weeks.

## Ethical Considerations

Ethical approval was obtained from the University Ethics Research Committee (no. 695-10). The study followed the Declaration of Helsinki Ethical Principles for Medical Research Involving Human Subjects (World Medical Association, 2013). The patients received both oral and written information about the research project, and they were informed they were free to withdraw without giving any explanation. Patients who volunteered gave their written consent. In cases or situations where patients were unable to consent due to critical illnesses, a present relative could give proxy consent. This was later followed up by the researcher, and the consent process was repeated to the patient when their health had improved enough to do so.

## Analyses

The results are based on two separate two-way analysis of covariance (ANCOVA), where the covariate consists of SAPS. First, the patient recovery data were analyzed using a 2 (mechanically ventilated, nonmechanically ventilated) × 2 (intervention room, ordinary room) ANCOVA. This analysis was performed in two separate analyses with the 6-month and 12-month follow-up questionnaire results as the dependent variables. Second, in the same way, a 2 (male, women) × 2 (intervention room, ordinary room) ANCOVA was performed. The dependent variables for all analyses were the self-reported recovery data at the 6- and 12-month follow-up, respectively. Both ANCOVAs were two-tailed with significance set at 5%. Findings of an interaction effect between the independent variables, room, gender, and mechanical ventilation, suggest patient recovery differed as a result of a combination of the room and these independent variables. A main effect of the ANCOVA would indicate either a group difference between the different conditions or a group difference between gender, patients who had been mechanically ventilated, and those who had not for the entire group of patients in the study. The dependent variable in these analyses was the self-reported recovery score. *t* tests were used for the means and standard deviations for the dependent variables. All statistical analyses were performed using Statistical Product and Service Solutions (SPSS) Statistics Version 25 (IBM, Armonk, New York).

## Results

A total of 381 patients were cared for in the two rooms between August 2012 and May 2014, and the questionnaires were sent to 169 of those (see Figure 6). The response rate was 60% (*n* = 101), and of these, 45 and 56 were cared for in the intervention room and ordinary room, respectively. There were 73 completed questionnaires from patients cared for in the intervention room (39 at 6 months’ postdischarge and 34 at 12 months) and 85 (46 at 6 months and 39 at 12 months) from the ordinary room. There were no significant differences between the two experimental groups in terms of age, gender, SAPS, and the number of mechanically ventilated patients (see Table 1). However, significant differences were revealed between the group of not participating patients and the group of participating patients. The participating patients were significantly older and had lower SAPS (see Table 4). Patients spent on average 98 hr in the intervention room (median = 48.00, interquartile range [IQR] = 85.50). In the ordinary room, they spent 118 hr on average (median = 72.00, IQR = 105.50). The results are presented for the dependent variable as an interaction between the type of respiratory ventilation and gender at both 6 and 12 months (for the means and standard deviation, see Table 5).

**Table 4.** Nonparticipating Versus Participating Patient Characteristics.

Characteristics	Nonparticipating Patients	Participating Patients	*p* Value
*N*	280	101	
Mean age (*SD*) ^a^	59 (22)	63 (14)	<.05
Number of men (%) ^b^	164 (59)	55 (54)	.63
Mean SAPS (*SD*) ^a^	55 (19)	49 (17)	<.01
Number of mechanically ventilated patients (%) ^b^	185 (66)	54 (53)	.05

*Note.* SAPS = Simplified Acute Physiology Score.

^a^ *p* Values are based on *t*-test comparisons of mean values. ^b^ *p* Values are based on χ^2^ comparisons of frequencies.

**Table 5.** Self-Reported Recovery at 6 and 12 Months Between Conditions.

Self-Reported Recovery	Intervention Room		Ordinary Room	
	*N*	*M* (*SD*)	*N*	*M* (*SD*)
6 Months	39	9.74 (4.33)	46	11.58 (4.38)
12 Months	34	8.88 (4.07)	39	10.90 (4.26)
Total	73		85	

### Main Effect of Different Rooms

The results revealed that the two conditions had no main effect at 6 months, *p* = .08, but at 12 months, there was a significant difference, *p* < .05, indicating that patient recovery was positively influenced for patients cared for in the intervention room (*M* = 8.88, *SD* = 4.07) than in the ordinary room (*M* = 10.90, *SD* = 4.26).

### Main Effect of Mechanical Ventilation

The analyses revealed that there was no main effect of mechanical ventilation at 6 months, *p* = .72, or at 12 months, *p* = .08, in regard to self-reported recovery.

### Main Effect of Gender

The data revealed no gender main effect at 6 months, *p* = .07, or at 12 months, *p* = .97.

### Interaction Effect Between Type of Respiratory Ventilation and Gender

There was no interaction effect between type of room and type of respiratory treatment at 6 months, *p* = .33, or at 12 months, *p* = .98. Neither was there an interaction effect for the type of room and gender at 6 months, *p* = .51, or at 12 months, *p* = .80. The analyses revealed that patient recovery did not differed as a result of a combination of the room and these independent variables.

## Discussion

A comparison between patients’ self-reported recovery scores between the two conditions revealed that those who had stayed in the intervention room reported a better recovery at 12 months but not at the 6-month mark. The result of environmental effects on patients’ recovery is supported by previous research. A study showed that recovery was enhanced by a multicomponent, nonpharmacological perioperative intervention in elderly cancer patients (*n* = 160; Guo et al., 2016). This intervention included additional teaching to the staff and familiarizing patients to equipment and the environment at a surgical ICU. Their study also involved clocks and calendars to keep patients orientated to the day and time. To maintain a good sleep–wake cycle, the room was illuminated by natural light during the daytime and low levels of artificial light at night. Blindfolds, acoustic earplugs, and minimized nursing procedures during the nights were also implemented. After surgery, patients in the intervention group had significant higher levels of melatonin and lower cortisol levels at night, significant lower levels on the Richmond Agitation–Sedation Scale and a lower incidence and shorter duration of postoperative delirium. Their recovery progress was measured by the Quality of Recovery 40 questionnaire. The results revealed significantly better recoveries for the intervention group. Furthermore, in a review study covering CR disruptions in ICU patients, it was reported that morning light decreased the prevalence of delirium and/or sleep disturbances in the acute medically ill (Oldham et al., 2016). There may be a link between lighting environments in a normal day and night rhythm and patients’ CRs, which were detected through higher levels of melatonin at night.

***A comparison between patients’ self-reported recovery scores between the two conditions revealed that those who had stayed in the intervention room reported a better recovery at 12 months but not at the 6-month mark.***

The reason for why there was a significant difference in patients’ recovery scores at 12 months’ postdischarge, but not at six, in relation to the two conditions is more speculative. The recovery process is fragile and not completely identified. Previous research (Dowdy et al., 2005) has reported that most patients perceived a mix of improvements and impairments in health-related QoL for up to 12 months after discharge.

To date, the ICU environment is designed for functionality and to avoid contamination (Thompson et al., 2012). This study’s concept has a holistic approach and focus on the interaction between people and their environment. The connection to nature is important. In ICU patient rooms, natural light in normal rhythms, nondisturbing sounds, green colors, and connections to the outdoor environment are important factors (Jonas et al., 2014; Minton & Batten, 2016). In a review, there was especially strong evidence between access to daylight and appropriate lighting with reduced depression (Ulrich et al., 2008). Similarly, there was a correlation between views of nature (requires daylight) and reduced pain and stress. Noise-reducing finishes reported strong evidence for less patient stress. There was a significant but weak correlation between lighting, daylight and noise with reduced medical errors, less pain, increased sleep, and increased patient satisfaction. Daylight and/or lighting was associated with fewer patient falls, less stress, and shorter length of hospital stays. Patients who received more sunlight reported significantly reduced time for weaning (Wise & Wallace, 2012).

## Limitations

As the CR is determined by melatonin levels, it would be beneficial to measure the participating patient’s melatonin regularly throughout the day and night to make the connection between patients’ CR and their recovery clearer. Only a few studies have evaluated environmental interventions in adult ICUs and their effects upon sleep or CRs. Moreover, most of the studies have small samples and produce inconsistent results (Li et al., 2011; Perras et al., 2007; Weiss et al., 2016). More full-scale intervention research is needed to determine the environmental effects of the caring practice in the ICU as the use and evaluation of various components may increase recovery in a nonharmful and relatively cost-effective way.

There were other changes in the intervention room on top of the cycled lighting system, such as different ceiling tiles and textiles, which could have changed the acoustic environments in the intervention room as well. However, the lighting intervention was considered to be the principal change in the room. In the literature, it is reported that normal CRs are supported by daylight and lighting that follow a natural pattern, mediated by the release of hormones (Bedrosian et al., 2016; Bonmati-Carrion et al., 2014). Noise is also able to disturb sleep and by extension the CR. However, the acoustic difference between the rooms was minimal with only a slightly better distinctness of speech and reverberation in the intervention room (Johansson, 2014).

Due to uncontrollable factors related to the ICU, such as patients’ special medical needs, it was not possible to do an entirely randomized study. The used consecutive sample reported as two groups of patients in different rooms with no significant differences in characteristics. The patients who completed the questionnaire differed in age and SAPS values compared to the excluded and nonparticipating group. Participating patients’ mean age was higher. Reasons for this may include that younger people were more often admitted to the ICU during the weekends due to intoxication or accidents related to intoxication and were, therefore, missed. Additionally, their ICU stays were generally shorter than 24 hr since they tended to recover faster. Perhaps older people are maybe more conscientious about their health and are, therefore, more likely to respond to health questionnaires. Participating patients reported lower SAPS values, indicating that the group of patients with the most severe illnesses were not able to participate. In the future, it would be important to include this group of patients, as they are the most vulnerable, and we need more knowledge about their situation to be able to improve their recovery.

Conducting research in the ICU is a challenge due to the complex situation with confounding variables. It is important in future research to develop and validate instruments investigating patients’ health in combination with their environment. The results from this study may be applicable to other similar contexts if the same lighting intervention is used. Further full-scale studies are necessary for the link between the environment and the patients’ recovery to become clearer.

## Conclusion

Patients’ self-reported recovery may have been supported by an environmental intervention, including a cycled lighting system, with a natural day and night rhythm, color, and location. The aim with the intervention was to stabilize patients’ CRs. Recovery is a fragile process and may be stabilized and sped up by having a normal CR supported by the lighting intervention. There were no interactions found between mechanical ventilation, gender, and the cycled lighting environment.

## Implications for Practice

- ICUs should keep lighting at low levels during the night and higher levels during the day and enable daylight to brighten up the patient room.
- When planning the ICU environment, great attention should be taken to allow natural daylight to light the room; for artificial lighting, the intensity, timing, and spectral distribution must be considered.
- A lighting environment that follows the day and night rhythm may support patient recovery.
- Longitudinal monitoring of patient recovery is crucial to understanding how ICU interventions aid recovery with longer term care.
